# Liver Hemangioma Might Lead to overestimation of Liver Fibrosis by Fibroscan; A Missed Issue in Two Cases

**DOI:** 10.5812/hepatmon.6685

**Published:** 2012-06-30

**Authors:** Seyed Hossein Aalaei-Andabili, Leila Mehrnoush, Shima Salimi, Mustafa Shafiei, Seyed Moayed Alavian

**Affiliations:** 1 Baqiyatallah Research Center for Gastroenterology and Liver Disease (BRCGL), Baqiyatallah University of Medical Sciences, Tehran, IR Iran; 2 Rezvan Medical Research Institute, Tehran, IR Iran

**Keywords:** Liver, Hemangioma, Fibrosis

## Abstract

**Background:**

The assessment of liver fibrosis is an important way for prediction of liver disease progression and patient’s prognosis. Liver stiffness measurement (LSM) is strongly associated with stage of liver diseases. overestimation of liver fibrosis in heart failure has been reported. We would like to introduce a new leading cause of liver fibrosis overestimation by presentation of two cases.

**Case Presentation:**

one case with right lobe hemangioma has an overestimation of liver fibrosis. The result completely changed when Fibroscan was performed in patient’s left lobe. Interestingly, another case with left lobe hemangioma had overestimation of fibrosis in her left lobe but, right lob Fibroscan was normal.

**Conclusion:**

We found that liver hemangioma may leads to overestimation of liver stiffness and the correct inspection of liver echogenicity before any interpretation of high liver stiffness is recommended. We suggest that patient with higher level of Fibroscan score repeat it in other sides of the liver. Also, they should be evaluated by sonography for ruling out of possible confounders such as hepatic hemangioma.

## 1. Background

Hemangioma is a benign, asymptomatic, and suddenly founded hepatic tumor [1]. Prevalence of hemangioma is high in general population [2]. Diagnosis of hemangioma is on base of liver imaging [1]. Effect of presence of hemangioma in the liver and misjudgment regarding liver stiffness by Fibroscan did not report yet. The assessment of liver fibrosis is an important variable for prediction of liver disease progression and patient’s prognosis [3]. Liver biopsy is known as gold standard of fibrosis determination; but, it is invasive and correlated with patient’s discomforts. Also, result of biopsy is depending on sampling skill, intra-observer and inter-observer variability [4]. Therefore, many researches were searched non-invasive method of liver fibrosis assessment. Fibroscan (FS) or transient elastography is a non-invasive and rapid method for liver stiffness measurement (LSM). LSM is strongly associated with stage of liver diseases [5]; although using the Fibroscan is impossible in subjects with ascites and is difficult in obese patients [6]. Over-estimation of liver fibrosis by Fibroscan has been reported at increased level of alanine aminotransferase (ALT) [7] and bilirubin, prolonged prothrombin time, severe hepatic congestion [8], heart failure, and cardiopulmonary congestion [9]. We would like to introduce a new leading cause of liver fibrosis overestimation by presenting two cases of hemangioma and necessity of correct inspection and finding the liver echogenicity before any interpretation.

## 2. Case Presentation

### 2.1. Case Number One

**Figure 1:**
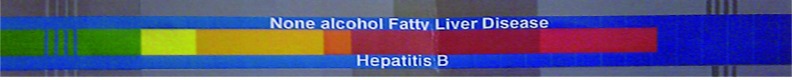
First Case Fibroscan Result From Hepatic Hemangioma Side

**Figure 2:**
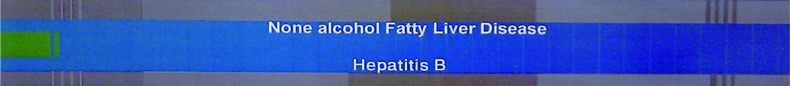
First Case of Fibroscan Result From Opposite Side of The Hepatic Hemangioma

The subject was a 34 years old female. She was known case of hepatic hemangioma from two years ago. Also, she had fatty liver and had come to our clinic for following up her problem by Fibroscan. The body mass index of patient was 23.7. In her first Fibroscan, median fibrosis score of her liver was 17.1 kilo Pascale (Kpa) which was compatible with F4 on Metavir histological index (Figure 1). Her cap score for liver steatosis was 201. The finding was unexpected; the patient was referred for liver sonography. Sonography confirmed her mild fatty liver, hepatic hemangioma (7.5×5.5 mm) in right lobe of liver, but there was no other abnormal finding. Liver function tests were normal. We tested autoimmune diseases and viral hepatitis markers for finding the reason; but all of them were normal. Based on our previously literature review, we thought hepatic hemangioma might leads to overestimation of liver fibrosis. So, we repeated Fibroscan (Echosens 502 device, France) from other sides; upper and lower to the hemangioma. Also, from her left lobe of liver by forth fold increasing of probe shuts. The findings was interesting, median fibrosis score was 4.4 that was compatible with F0 (Figure 2) but cap score of liver steatosis was near to our previous finding (210 dbl/m). We confirmed our guess, but more evidences were required. In the second Fibroscan we attend more to liver echogenicity and we excluded the mixed echogenicity parts for shutting.

### 2.2. Case Number Two

**Figure 3:**
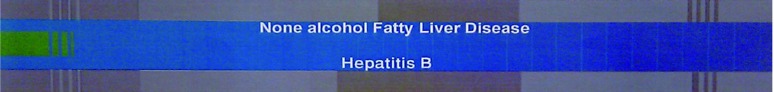
Second Case Fibroscan Result From Opposite Side of the Hepatic Hemangioma

**Figure 4:**
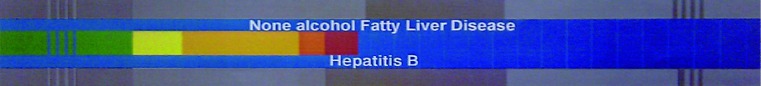
Second Case Fibroscan Test Result From Hepatic Hemangioma Side

**Figure 5:**
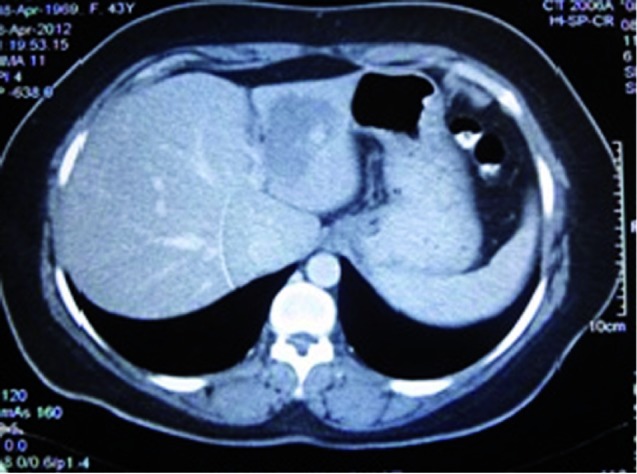
Second Case Left Lobe Hepatic Hemangioma

Interestingly, another patient came to our office for her fatty liver treatment follow up. We reviewed her medical documents for our new decision. We found that she is a known case of hepatic hemangioma. We demanded her to accept Fibroscan test. There was no problem for Fibroscan performance and patient’s BMI was 24.1. Then, Fibroscan was done. Her fibrosis assessment by standard approach (right lobe) was normal and stiffness score of her liver was 5.6 Kpa which was equal with F0-F1 (Figure 3). Also, her cap score for liver steatosis was 323 dbl/m. For evaluation of hepatic hemangioma effect in Fibroscan results, we repeated the procedure by forth fold increasing of probe shuts in left lobe of liver that the hemangioma was exist. Interestingly, result of Fibroscan was the same as cirrhotic patients; Fibroscan score was 11.8 Kpa (F3-F4) (Figure 4). But, the cap score for liver steatosis did not change much more and was 335 dbl/m. Sonography and CT scan (Figure 5) have confirmed hepatic hemangioma (50-60 mm) in left lobe of patient’s liver. Also, cavernous hemangioma has been reported in her liver biopsy.

## 3. Discussion

We found that hepatic hemangioma is a leading cause of liver fibrosis overestimation. Overestimation of liver fibrosis has been reported in the presence of heart failure [9]. Effect of heart failure on liver has been understood [10]. This effect is known as cardiac hepatopaty [11]. Although chronic heart failure can result in irreversible liver injury and cirrhosis [12] but the impact of this finding is not well known. However, liver congestion is considered as main induced factor of over-estimation. Also, alkaline phosphatase (ALKP) was increased in heart failure induced overestimation [9]; but, in our finding all laboratory tests were normal. In a study, LSM of patients have decreased significantly after improvement in heart failure degree. On the other hand, in an animal model, central venous pressure was known as controller of the liver stiffness [13]. Fibroscan performance in hemangioma side overestimates liver fibrosis and makes false positive results. Since, higher level of Fibroscan score is associated with higher level of mortality even in absence of liver diseases [14]; a precise and exact determination of liver fibrosis is very important. We suggest that Fibroscan should be done on the opposite side of hemangioma in known patients of Hemangioma and the correct inspection of liver echogenicity before any interpretation of high liver stiffness is recommended. Also, it is better to perform Fibroscan in various sides of the liver for all patients undergoing Fibroscan test. In addition, sonography should be considered as further evaluation approaches in patients with high Fibroscan score.
